# Correlation between central stromal demarcation line depth and changes in *K* values after corneal cross-linking (CXL)

**DOI:** 10.1007/s00417-018-3922-z

**Published:** 2018-02-23

**Authors:** Niklas Pircher, Jan Lammer, Stephan Holzer, Andreas Gschließer, Ruth Donner, Stefan Pieh, Gerald Schmidinger

**Affiliations:** Department of Ophthalmology, Medical University of Vienna, Waehringer Guertel 18-20, 1090 Vienna, Austria

**Keywords:** Corneal cross-linking (CXL), Demarcation line, Keratometry, Riboflavin

## Abstract

**Purpose:**

A stromal demarcation line (DL) after corneal cross-linking (CXL) has lately been suggested as a surrogate parameter for the success of CXL. The aim of this study was to investigate the correlation between depth of the central DL 1 month and the change in *K* values 12 months after CXL.

**Methods:**

Treatment-naive subjects with keratoconus were treated using an accelerated CXL protocol [A-CXL(9*10)]. Depth of the DL/relative depth of the DL (DL%) was measured using Visante OCT imaging 1 month postoperatively (OP). *K*_max_/*K*_2.5_ (preOP) and change in *K*_max_/*K*_2.5_ (preOP − 12 months postOP) were assessed using corneal tomography (Pentacam HR, Oculus GmBH).

**Results:**

Forty eyes were treated following the A-CXL(9*10). The mean DL depth was 200 ± 99 μm (range 71 to 479)/mean DL% = 42.70 ± 20.00% (range 17–90). There was no statistically significant correlation between stromal depth of the DL and change in *K*_max_ or *K*_2.5_, respectively (Spearman rho DL/∆*K*_max_ − 0.14 and DL/∆*K*_2.5_ − 0.14). Between DL% and the changes in maximum *K* values or *K*_2.5_, no statistically significant correlation was found as well (Spearman rho DL%/∆*K*_max_ − 0.10 and DL%/∆*K*_2.5_ − 0.19). Mean change in *K*_max_ after 12 months was − 0.68 ± 2.26 diopters (D) (median − 0.35 D) and − 0.82 ± 1.6 D (median − 0.65 D) for *K*_2.5_ (*p* = 0.07; *p* = 0.02).

**Conclusions:**

No statistically significant correlation was found between the stromal central depth of the DL and any outcome parameter for CXL after 12 months. Therefore, the interpretation of the DL as a predictive parameter for the effect of the procedure may not apply.

## Introduction

The corneal stromal demarcation line (DL) and its central depth has frequently been suggested as a possible predictive parameter for the effectiveness and success of corneal cross-linking (CXL) [1, 2].

The appearance of a DL after CXL following the standard Dresden protocol [S-CXL(3*30)] was first reported by Seiler and Hafezi in 2006. This stromal line was detected 2 weeks after treatment by slit lamp examination at approximately 300-μm depth at the center of the cornea [2]. Differences in refractive indices or reflection properties between treated and untreated corneal stromas have been postulated as a possible explanation for the occurrence of the demarcation line, representing the transition zone between anterior cross-linked and posterior non-cross-linked corneal tissues [2]. Furthermore, confocal microscopy enabled the visualization of a transition area, where an oedematous zone characterized by keratocyte apoptosis merges with a zone with common keratocyte count [3]. However, the exact patho-physiological reason for the development of the DL remains to be elucidated. In addition to confocal microscopy and slit lamp examination, the demarcation line can also be quantified by anterior segment optical coherence tomography (AS-OCT) [4, 5]. Current manufacturer’s software kits enable the precise measurement of the depth of the demarcation line in the central B-scan of the cornea [4]. A shallower DL has recently been observed in an accelerated corneal cross-linking [6].

The aim of this study was to investigate the association between the mean depths of the DL in the center of the cornea 1 month postoperatively and changes in mean *K* values after 1 year.

## Methods

**Fig. 1:**
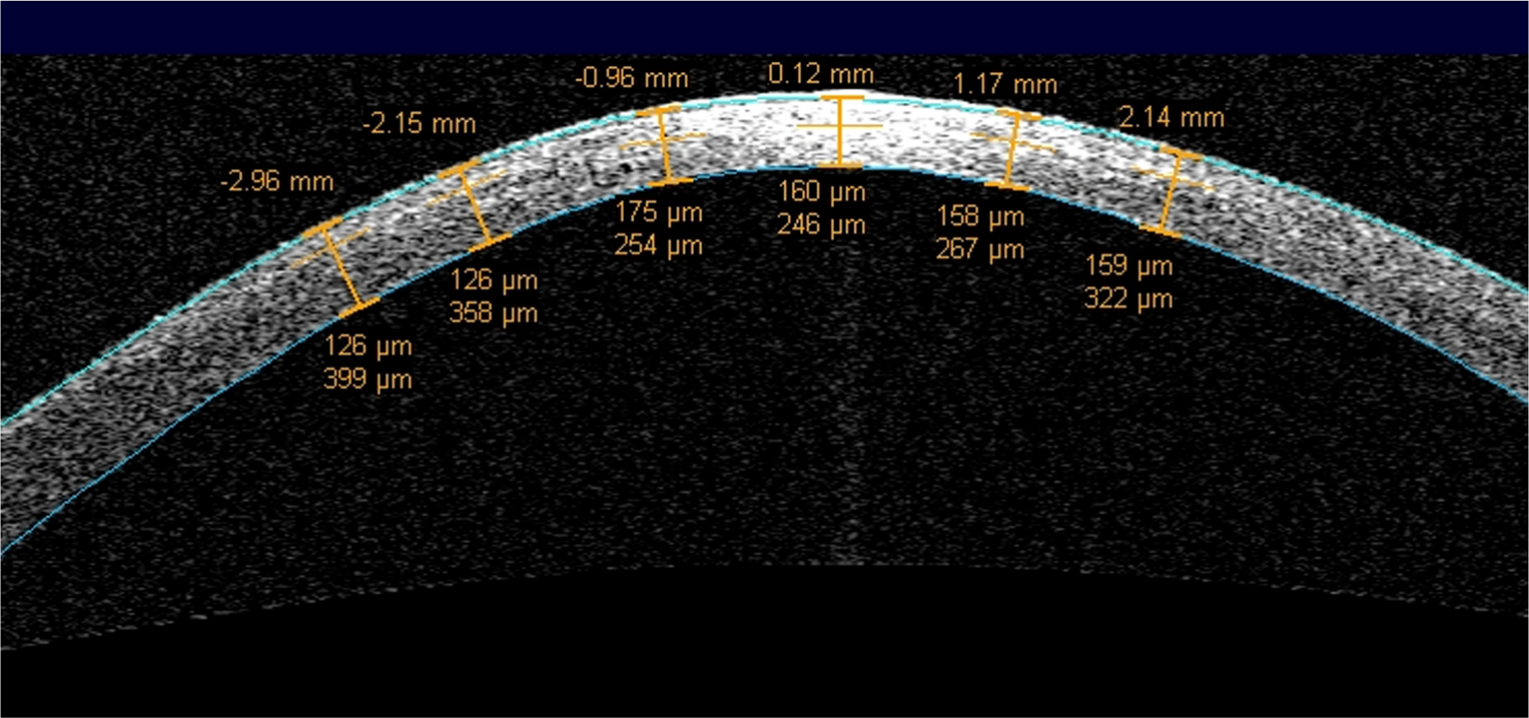
The depth of the DL was measured as the distance between corneal epithelium and the hyperreflective tissue

The investigations and measurements in this retrospective study were performed in accordance with the Declaration of Helsinki. The local ethics committee approved the protocol of this retrospective trial (EK Nr. 2225/2015). Patients were informed about the procedure and its potential risks. Prior to any surgical intervention, their written informed consent was obtained in accordance with institutional and legal requirements. Patient files between 2014 and 2016 of consecutive patients of our outpatient department with progressive keratoconus, which were scheduled for CXL, were evaluated. Progression was defined as an increase of more than one diopter in the maximum *K* value within the last year. A single surgeon (G.S.) performed all surgical procedures using a protocol for accelerated CXL [A-CXL(9*10)] which was conducted as follows: 10 min riboflavin with HPMC [Vibex Rapid] soaking, removing of the riboflavin film with physiological salt solution before irradiation, 10-min irradiation with the UVX-2000 (9 mW/cm^2^), riboflavin-dropping two times during irradiation. Postoperatively, the patients received a therapeutic contact lens, which was left in place until epithelial healing. Patients received antibiotic eye drops for 1 week and antiinflammatory/lubricating drops for 4 weeks. Depth of the DL was measured using Visante AS-OCT imaging (Carl Zeiss Meditec Inc.) 1 month postoperatively. Only the high-resolution scan of the horizontal meridian was used for the assessment of the DL. The depth of the central DL was measured using the flap tool as provided by the manufacturer’s software package as the distance from the corneal epithelium to the hyperreflective DL (Fig. 1). The relative depth of the central DL (in % of the total central corneal thickness) was determined at the same time point (DL%). Furthermore, postoperative examinations included corneal tomography (Pentacam HR tomography, Oculus GmbH, Germany) and slit lamp examination 1, 3, 6, and 12 months after CXL. Changes in mean *K*_max_ and in mean keratometric values in a 2.5-mm circular zone around the *K*_max_ (*K*_2.5_) were detected using Scheimpflug tomography and the software tool Corneal Power Distribution Display by the Pentacam. Only patients that were available for all follow-up visits were included. Visual acuity was measured as a safety parameter at subsequent time points but was not included in analysis at follow-up due to its high variability in keratoconus.

## Statistical analysis

Spearman correlation coefficients were calculated to assess the correlation between the depth of the DL in the center of the cornea at the 1-month follow-up visit and the change of mean *K* values after 12 months. To prove the statistical significance, an iterative statistical simulation was performed to calculate the probability to observe a Spearman *r* of > 0.3 if no correlation is expected (Spearman *r* = 0.0). Correlations were also drawn as scatter plots. To explore normal distribution, a Shapiro-Wilk analysis was performed. Continuous variables are given as the mean ± standard deviation (SD) + bootstrap confidence intervals or median. The Wilcoxon signed-rank test was used when comparing the differences for *K*_max_ and *K*_2.5_ as these data differed from normal distribution in the Shapiro-Wilk test. The level for statistical significance was set as less than 0.05. For statistical analysis, SPSS (version 23) was used.

## Results

Forty eyes of 40 patients met the inclusion criteria for this trial within the defined time frame. All eyes were treated according to A-CXL(9*10) as described above. Sixty-seven percent (*n* = 27) were male and 33% (*n* = 13) were female patients. The mean age was 27 years ± 8 (range 18 to 47). In all the patients, epithelial closure was observed after a maximum of 4 days. The Shapiro-Wilk test showed that the collected data for the DL were normally distributed (*p* > 0.05), but the collected data for *K*_max_ and *K*_2.5_ were not normally distributed (*p* < 0.05).

**Fig. 2:**
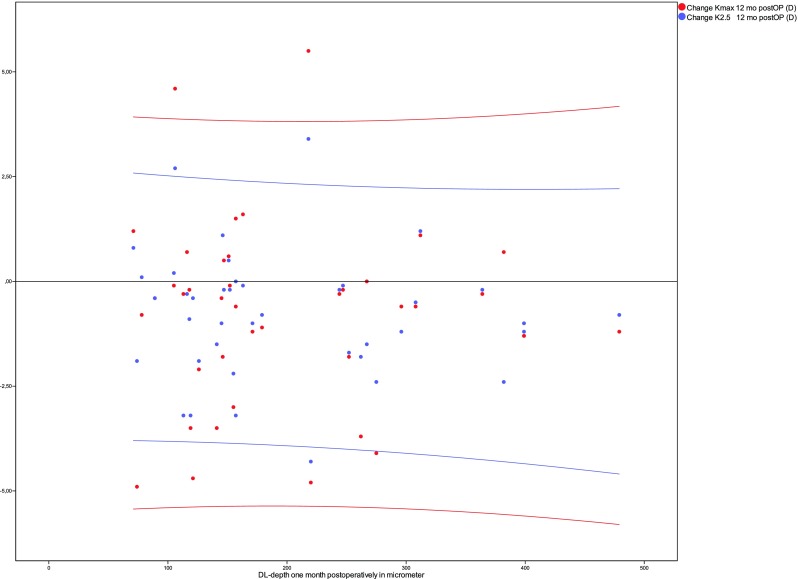
No statistically significant correlation was found between the depth of the central stromal DL after 1 month (*X*-axis) and changes in *K* values after 12 months (*Y*-axis)

**Fig. 3:**
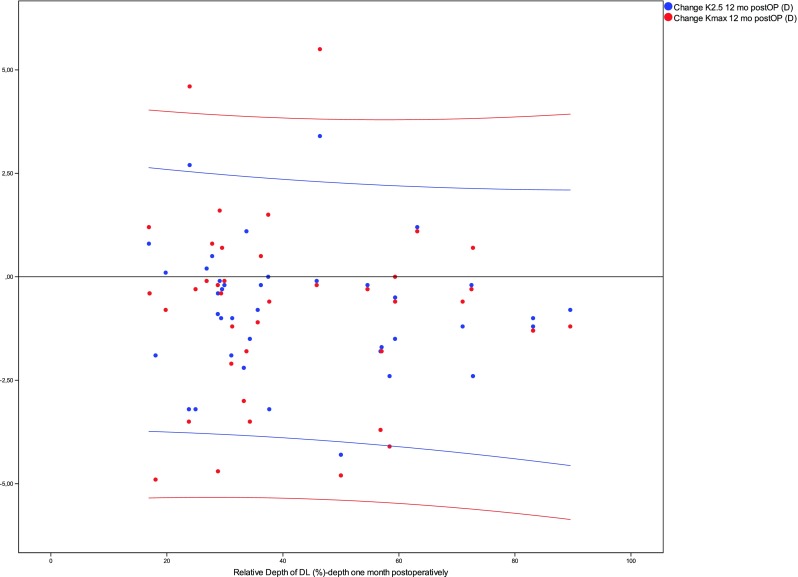
Between the relative depth of the central stromal DL after 1 month (*X*-axis) and the changes in *K* values (*Y*-axis), no statistical significant correlation was found

**Fig. 4:**
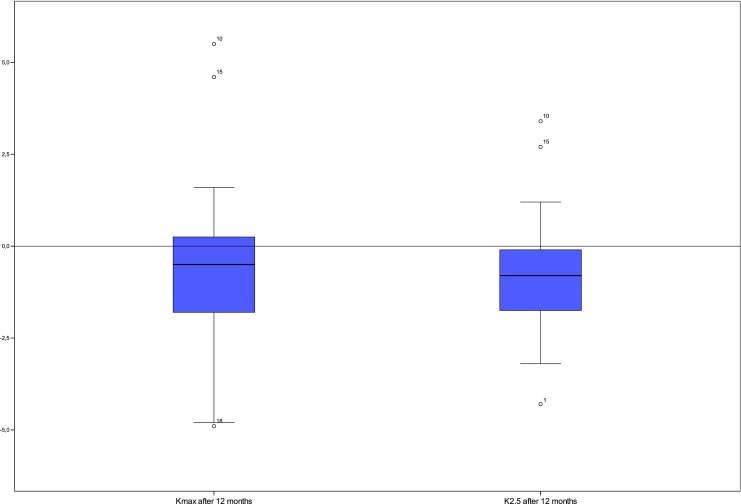
*K*_max_ and *K*_2.5_ showed a median of − 0.50 and − 0.80 D, respectively. This was a statistically significant finding for both values

The appearance of a stromal demarcation line was observed in all the eyes 1 month postoperatively and showed a mean central stromal DL of 200 ± 99 μm (range 71 to 479). The mean DL% was 42.7 ± 20.0 (range 17–90). A statistically significant correlation between the postoperative depth of the central stromal DL and changes in maximum *K* values or *K*_2.5_ 1 year after CXL was not found (Spearman rho DL/∆*K*_max_ − 0.14 and DL/∆*K*_2.5_ − 0.14, Fig. 2). Between the relative depth of the central stromal DL and the changes in maximum *K* values or *K*_2.5_, no statistically significant correlation was found as well (Spearman rho DL%/∆*K*_max_ − 0.10 and DL%/∆*K*_2.5_ − 0.19; Fig. 3). As the smallest clinically relevant effect, a correlation coefficient of 0.3 was chosen. Based on a simulation (with *N* = 10,000 iterations) with a sample size of *N* = 40, we found that the chance to observe a Spearman correlation coefficient of greater or equal to 0.3, if the true correlation coefficient is 0, is approximately 0.03. Changes in mean *K* values and levels of significance are given in Fig. 4. Preoperative mean *K*_max_ was 56.72 ± 7.62 diopters (D) (CI 54.49–59.15), increased to 57.20 ± 7.64 D after 1 month, and decreased thereafter to 56.70 ± 7.24 D at the 3-month visit, 56.07 ± 7.13 D at the 6-month visit, and 55.95 ± 7.55 D (CI 53.77–58.29) after 1 year. *K*_2.5_ was 52.00 ± 5.35 D (CI 50.40–53.68) before CXL and 51.20 ± 5.57 D (CI 49.58–52.94) at the 1-year follow-up visit. Six eyes (15%) showed an increase in *K*_max_ of more than one diopter and four eyes (10%) showed an increase of more than one diopter in *K*_2.5_ after 1 year (Table 1).

**Table 1:** DL characteristics of eyes that showed an increase in *K*_max_ or *K*_2.5_ of more than 1 D after 12 months

Patient	DL depth (μm)	*K*_max_ (D)	*K*_2.5_ (D)
No. 05	146	− 1.8	+ 1.1
No. 10	218	+ 5.5	+ 3.4
No. 12	71	+ 1.2	+ 0.8
No. 15	106	+ 4.6	+ 2.7
No. 24	157	+ 1.5	0.00
No. 28	163	+ 1.6	− 0.10
No. 31	312	+ 1.1	+ 1.2

## Discussion

Common to several earlier CXL studies is the assumption that the occurrence of the stromal demarcation line and its depth act as surrogate parameters for the effect and success of CXL [2, 6, 7]. To conclude that the depth of the stromal DL directly correlates with the outcome of CXL seems reasonable, given that it represents the transition zone between cross-linked and non-cross-linked tissues. Today, topographical stabilization (*K*_max_ or *K*_2.5_, respectively) counts as success and an increase in Young’s modulus after CXL can only lead to a stabilization of the cone. Eyes treated with A-CXL(9*10) as used in our trial showed a shallower demarcation line than usually seen in eyes treated with the standard Dresden protocol [6]. However, eyes with an increase in maximum *K* values showed variable depths of the DL which only reinforce the absence of a statistical correlation between DL and changes in *K* values (Table 1). To our knowledge, statistical correlations between the DL and changes in parameters such as *K*_max_ or *K*_2.5_ have only been evaluated to date with a small case series [8]. In the current trial, we did not find a statistical correlation between DL depth and the changes in *K* values. Based on the Bunsen-Roscoe law, which describes the reciprocal relationship between both the total amount of irradiation intensity and that of photochemical reaction, an increase of the radiant energy has been proposed to enable a shorter UV irradiation duration without minimizing the effect of CXL [9]. Following this hypothesis, accelerated cross-linking protocols with higher irradiation intensities and shorter irradiation times have been introduced [10]. The equivalence of S-CXL(3*30) and accelerated cross-linking protocols has recently been questioned, and one study showed a superior efficacy of the standard Dresden protocol compared to an accelerated protocol. In the said study, a shallower DL was mentioned in eyes treated according to an A-CXL(9*10), and in fact, an increase in *K*_max_ was found [6]. However, according to our results, the central stromal depth of the DL does not correlate with commonly used outcome parameters at all. Doubtless, the depth of the DL and the changes in *K* values as found in our study are certainly less distinct than results usually achieved with the standard Dresden protocol, but it seems that unknown factors other than the depth of the DL might influence the outcome of CXL. In our opinion, several parameters could influence the depth of the DL. Newer UV-light sources with a flat beam profile (KXL, Avedro) or “donut-shaped” beam profile (UVX-2000, Innocross) have been developed to obtain an optimized distribution of the energy across the corneal surface [11]. The depth of the DL could be determined by the beam profile of the UV source used for CXL [12]. The UV-X 1000 has a Gaussian beam profile and higher UV-A intensities are provided in the center of the beam with a gradual decline of the irradiation intensity towards the periphery [12]. This could lead to a deeper central demarcation line in patients treated with this device, as shown by Brittingham et al., who used different UV sources with identical soaking time (20 min) and riboflavin solutions [6].

In addition, new riboflavin solutions with HPMC (hydroxylpropyl methylcellulose) instead of dextran found its way into CXL treatment. Earlier studies mentioned a hypothetical explanation for a shallower DL in rapid protocols in a limited diffusion rate due to a decreased irradiation time during the CXL procedure [13]. However, according to literature, HPMC significantly increases the penetration of topical fluorescein as an ophthalmic carrier. That may provide the evidence to suggest that HPMC also increases the diffusion rate compared to the standard riboflavin [14]. Furthermore, a recent study showed comparable riboflavin gradients when comparing 30-min application of riboflavin-dextran and 10-min application of riboflavin-HPMC [15]. Given that Vibex Rapid ensures the same diffusion rate during 10 min as riboflavin solutions with dextran during 30 min [S-CXL(3*30)], the shallower DL as found in our trial must have another cause. By using iso-osmolar riboflavin solutions with HPMC, corneal thinning can be avoided, an effect commonly observed when using riboflavin solutions with 20% dextran [16–18]. The dehydration and hence corneal thinning induced by riboflavin solutions with 20% dextran as usually used in the standard Dresden protocol most likely explains the deeper demarcation line so commonly observed with this protocol [5]. Still, as shown in our trial, the depth of the DL does not indicate success of the treatment. There may be evidence that shorter irradiation times negatively influence the polymerization reactions due to insufficient oxygen diffusion [19]. The Bunsen-Roscoe might not apply to the CXL procedure and a loss of efficacy must be anticipated with higher UV-light intensities [13]. This could be one explanation why our study showed a worse outcome compared to common results with the S-CXL(3*30). It should be mentioned that one major weakness of this study is its retrospective design which by nature cannot eliminate possible confounders like age, gender, preoperative *K* values, and comorbidities like atopic diseases. In conclusion, this study showed that the widely proposed hypothesis of a correlation of the demarcation line depth and CXL outcome does most probably not apply, at least when using the herein described protocol. No correlation was found between the depth of the DL and the change in *K* values.
